# Serum Tumor Markers Combined With Clinicopathological Characteristics for Predicting MMR and KRAS Status in 2279 Chinese Colorectal Cancer Patients: A Retrospective Analysis

**DOI:** 10.3389/fonc.2021.582244

**Published:** 2021-06-17

**Authors:** Ning Zhao, Yinghao Cao, Jia Yang, Hang Li, Ke Wu, Jiliang Wang, Tao Peng, Kailin Cai

**Affiliations:** ^1^ Department of Gastrointestinal Surgery, Union Hospital, Tongji Medical College, Huazhong University of Science and Technology, Wuhan, China; ^2^ Department of Gastrointestinal Surgery, The Central Hospital of Wuhan, Tongji Medical College, Huazhong University of Science and Technology, Wuhan, China; ^3^ Department of Pancreatic Surgery, Union Hospital, Tongji Medical College, Huazhong University of Science and Technology, Wuhan, China

**Keywords:** kirsten rat sarcoma viral oncogene, mismatch repair proteins, clinicopathological characteristics, serum tumor markers, prediction

## Abstract

Although serum tumor markers (STMs), clinicopathological characteristics and the status of KRAS and MMR play an important role in optimizing the treatment and prognosis of colorectal cancer, their interrelationships remain largely unknown. A retrospective analysis of 2279 patients who tested for KRAS and MMR status, and STM measurements prior to treatment over the past four years was conducted. Of the 784 patients tested for KRAS and 2279 patients tested for MMR status, KRAS mutations and dMMR were identified in 276 patients (35.20%) and 177 patients (7.77%), respectively. Logistic regression analysis demonstrated that right colon, well and moderate differentiation and negative CA19-9 were independent predictors for KRAS mutations. The ROC curve yielded an AUC of 0.609 through the combination of these three factors. Age < 65 was an independent predictive factor for dMMR, along with tumor size > 4.6 cm, right colon, poor differentiation, harvested lymph nodes ≥ 22, no lymph node metastasis, no perineural invasion, negative CEA and positive CA72-4. When the nine criteria were used together, the AUC was 0.849. In summary, both STMs and clinicopathological characteristics were found to be significantly associated with the status of KRAS and MMR. The combination of these two factors possessed a strong predictive power for KRAS mutations and dMMR among CRC patients.

## Introduction

Colorectal cancer (CRC) is the third most common type of cancer and the second leading cause of cancer-related death worldwide (1). CRC imposes a substantial burden on the healthcare system, with the direct costs of CRC accounting for close to 10% and 12% of all direct cancer-related costs across the European Union (2) and the United States (3), respectively. It has been estimated that more than 20% of patients present with metastatic CRC (mCRC), and approximately half of patients with localized CRC will develop metastases (4). In the majority of mCRC, tumor lesions tend to be unresectable, and chemotherapy is recommended to prolong survival and improve symptoms. Fluoropyrimidine-based chemotherapy regimens and monoclonal antibodies directed against epidermal growth factor receptor (EGFR) are approved for first-line treatment of the disease. Molecular testing for KRAS and mismatch repair (MMR) status are mandatory to optimize the choice and sequencing of therapy (5).

Kirsten rat sarcoma viral oncogene (KRAS) is located downstream of EGFR signals, and KRAS mutations lead to its constitutive activation (6), which makes advanced colon cancer less responsive to anti-EGFR monoclonal antibodies such as cetuximab and panitumumab (7, 8). Mismatch repair (MMR) proteins are responsible for length alterations in microsatellites as they correct strand alignment and base matching errors during DNA replication (9). CRC patients with mismatch repair deficiency (dMMR) are not only likely to have a better prognosis (10), higher incidence of Lynch syndrome (11) and a high response to immune checkpoint blockade, but are less likely to benefit from 5-FU-based chemotherapy (12, 13). However, translating genetic testing into routine clinical practice is frequently hindered by many barrier factors, such as the high cost of testing and the need for specialized clinical laboratories (14–16). These difficulties are particularly obvious in developing countries, especially in county-level hospitals. Therefore, there is an urgent need to develop a convenient, non-invasive and cost-effective modality to identify appropriate candidates for genetic testing.

Previous studies have demonstrated that serum tumor markers (STMs) and clinicopathological characteristics are both important prognostic factors as well as indicators of the therapeutic effect and recurrence risk in patients with CRC (17–19), while their association with KRAS and MMR status is largely unknown. In this study, we explored the predictive value of STMs in combination with clinicopathological indicators for KRAS and MMR status across East Asian CRC patients.

## Methods

### Study Design and Patient Cohort

From January 6, 2016, through December 10, 2019, a total of 5457 patients were diagnosed with CRC in our centre. All patients were subjected to thorough history taking, and their information was collected from “Biological big data platform for individualized diagnosis and treatment of gastrointestinal cancer” (national software copyright 2019SR1267841). This study protocol was approved by the ethics committee of our college.

**Figure 1:**
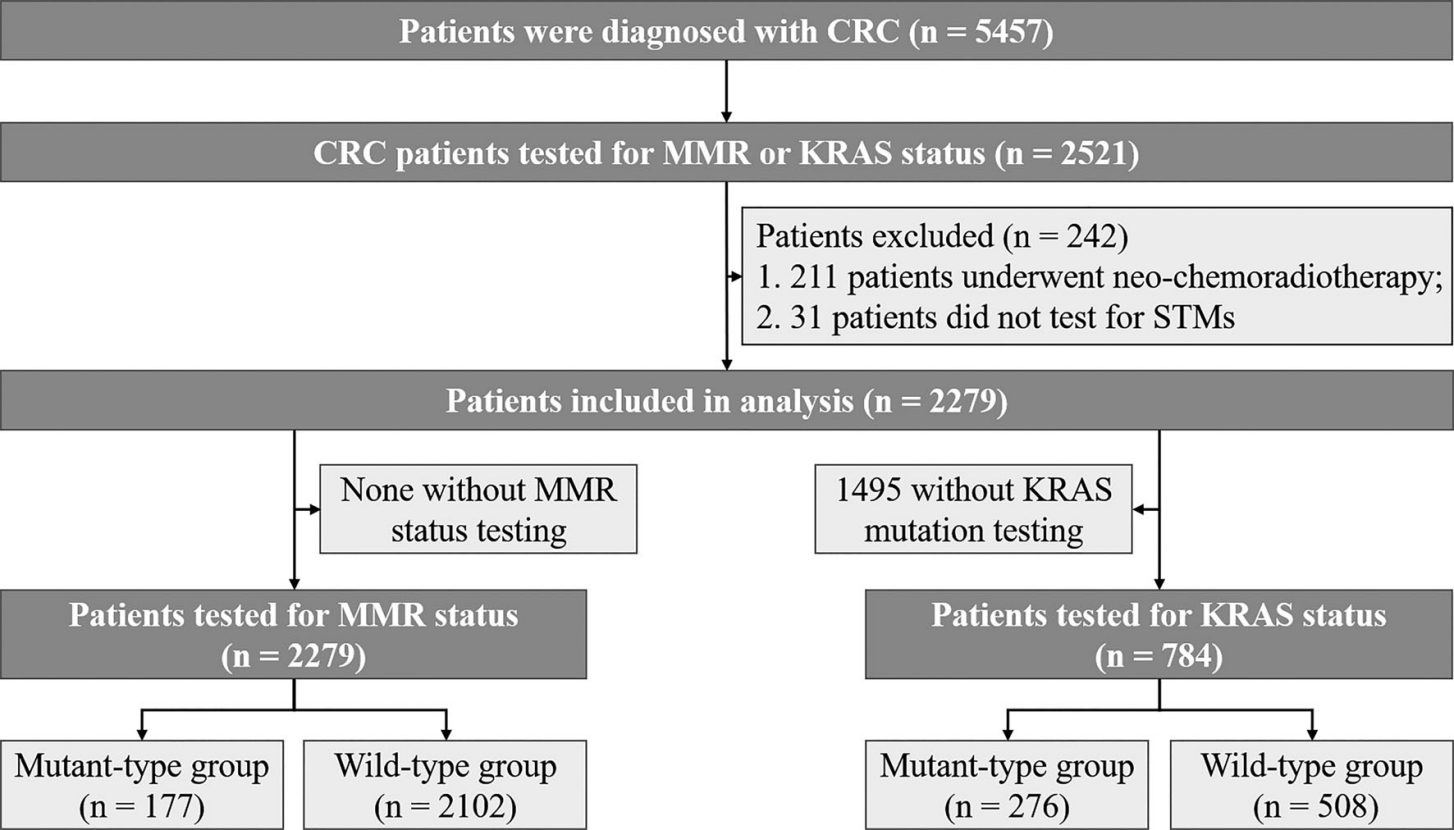
Study design and algorithm of patient selection.

A flow diagram for screening the eligible CRC patients is presented in **Figure 1**. A total of 2521 patients with MMR or KRAS testing were identified. A total of 242 patients with the following conditions were excluded from the study: (1) 211 patients underwent neo‐chemoradiotherapy before KRAS and MMR status detection; (2) 31 patients did not have data for STMs. Tumor stage was classified according to the 8th edition of the American Joint Committee on Cancer Staging System.

### KRAS Mutation Analysis

**Figure 2:**
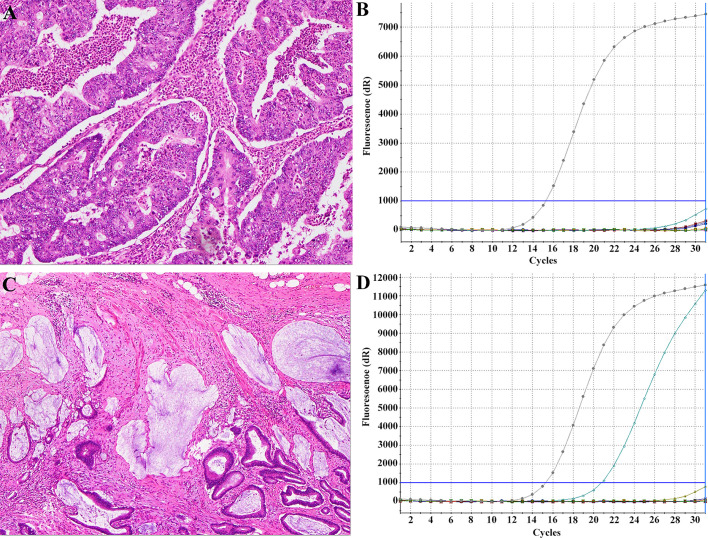
Representative histological images with KRAS mutant or wild-type CRC. Findings of a 53-year-old man with KRAS-wild type CRC **(A)** and a 45-year-old woman with KRAS-mutant type CRC **(C)** with haematoxylin-eosin staining showing histological type and the ARMS method **(B, D)** demonstrating KRAS status type.

The primers for the amplification and Sanger dideoxy chain termination sequencing of KRAS gene exon 2 were forward 5′‐GTCCTGCACCAGTAATATGC‐3′ and reverse 5′‐ATGTTCTAATATAGTCACATTTTC‐3′ for exons 3 and 4. Polymerase chain reaction (PCR) was performed using 100 ng of genomic DNA as a template. Each mixture contained 10 pmol of each primer. The reactions were performed in a total volume of 31.5 μL. The amplification reaction was as follows: an initial denaturing cycle of 95°C for 5 min; 35 cycles of 94°C for 25 s, 58°C for 25 s, 72°C for 25 s; and a final extension cycle at 72°C for 10 min. The PCR products were then purified and subjected to direct sequencing using an automatic sequencer (ABI‐3730 DNA Sequencer; Life Technologies, CA). Tumors with any KRAS mutations were classified as mutant KRAS, whereas the rest were classified as wild-type KRAS. Representative histological images of two patients with KRAS mutant or wild-type CRC are shown in **Figure 2**.

### Immunohistochemical Analysis of MMR Status

Immunohistochemical staining was performed by the streptavidin-biotin-peroxidase detection method. First, CRC tissues fixed with 4% formaldehyde and embedded with paraffin were cut into 5 µm thick slices and then fixed onto glass slides. After rehydration with ethanol and microwave antigen retrieval, tissue sections were labelled with anti-MSH2 antibody (A1121, 1:200), anti-MSH6 antibody (A3177, 1:100), anti-MLH1 antibody (A0254, 1:100) or anti-PMS2 antibody (A6947, 1:100) overnight at 4°C. After washing with PBS, slides were incubated with the specific HRP-conjugate antibody at 37°C for 10 min, cleaned with cold PBS and treated with peroxidase-conjugated biotin streptavidin complex for 10 min. Finally, staining was performed with DAB and counterstaining was performed with haematoxylin. Binary interpretation was used to determine whether MMR was deficient or proficient as follows: tumors displaying loss of expression of one or more MMR proteins were considered to be dMMR, whereas tumors with intact MMR proteins were classified as pMMR.

### STMs Measurements

Prior to any anticancer treatment, blood samples were obtained through peripheral venipuncture, and STMs were detected by a commercial chemiluminescence immunoassay kit (Abbott Laboratories, I4000, America). The detected STMs included carcinoembryonic antigen (CEA), alpha fetoprotein (AFP), squamous cell carcinoma antigen (SCC), neuron-specific enolase (NSE), carbohydrate antigen (CA) 72-4, CA 125, CA 15-3, CA 19-9, ferritin (FERR) and soluble fragment of cytokeratin 19 (CYFRA21-1), which had threshold values of 5 µg/L, 8.78 µg/L, 1.5 ng/ml, 16.3 µg/L, 6.9 U/ml, 35 U/ml, 31.3 U/ml, 37 U/ml, 275 µg/L (male) or 204 µg/L (female), 2.5 ng/ml, respectively. Tumor marker values above these thresholds were considered positive.

### Statistical Analysis

Statistical analysis was performed using SPSS 23.0 (SPSS Inc., Chicago, IL, USA). Data are presented as numbers and percentages for categorical variables, and continuous data are expressed as the mean ± standard deviation (SD), unless otherwise specified. Patient characteristics were compared using t tests for continuous variables and X^2^ or Fisher exact tests for categorical variables. All candidate predictors with a P < 0.05 in univariate analysis were included in a multivariate logistic regression model. The discrimination ability of individual and combined factors was measured by the area under the ROC (receiver operating characteristic) curve (AUC). A value of P < 0.05 was considered significant.

## Results

### Patient Clinical Characteristics

Among the 2279 recruited CRC patients, the number of participants tested for KRAS and MMR was 784 and 2279, respectively. The characteristics are summarized based on whether the patients were tested for KRAS or MMR (**Table 1**). Of the 2279 patients tested for MMR status, dMMR were identified in 177 patients (7.77%). Among the 784 CRC patients tested for KRAS status, 276 (35.20%) patients presented KRAS mutations: 42.39% (117/276) in codon 12 (most commonly G12D and G12S), 21.38% (59/276) in codon 13 (most commonly G13D), 23.19% (66/276) in both codon 12 and 13, 3.62% (24/276) in codon 61, 8.70% in both codon 117 and 146.

**Table 1:** Associations of clinical characteristics with MMR and KRAS status in all participants.

	MMR			KRAS		
	pMMR	dMMR	P-Value	Wild-Type	Mutant-Type	P-Value
**Age (years)**			0.008			0.205
<65	1508 (91.28)	144 (8.72)		364 (66.30)	185 (33.70)	
>=65	594 (94.74)	33 (5.26)		144 (61.28)	91 (38.72)	
**Gender**			0.914			0.43
Female	840 (92.31)	70 (7.69)		197 (63.14)	115 (36.86)	
Male	1262 (92.18)	107 (7.82)		311 (65.89)	161 (34.11)	
**Histology type**			0.005			0.005
non-adenocarcinoma	515 (89.41)	61 (10.59)		114 (56.44)	88 (43.56)	
adenocarcinoma	1587 (93.19)	116 (6.81)		394 (67.70)	188 (32.30)	
**Tumor size**			<0.001			0.447
<=4.6cm	1364 (96.40)	51 (3.60)		288 (63.58)	165 (36.42)	
>4.6cm	738 (85.42)	126 (14.58)		220 (66.47)	111 (33.53)	
**Tumor location**			<0.001			0.009
Right	448 (79.72)	114 (20.28)		142 (57.96)	103 (42.04)	
Left	1654 (96.33)	63 (3.67)		366 (67.90)	173 (32.09)	
**Degree of differentiation**			<0.001			0.003
Low	263 (86.80)	40 (13.20)		94 (77.69)	27 (22.31)	
Moderately and highly	1719 (94.14)	107 (5.86)		382 (63.14)	223 (36.86)	
**T stage**			0.025			0.229
I/II	385 (95.06)	20 (4.94)		72 (70.59)	30 (29.41)	
III/IV	1717 (91.62)	157 (8.38)		436 (63.93)	246 (36.07)	
**Harvested lymph nodes**			<0.001			0.842
<22	1508 (95.38)	73 (4.62)		318 (65.16)	170 (34.84)	
>=22	594 (85.10)	104 (14.90)		190 (64.19)	106 (35.81)	
**Lymph nodes metastasis**			<0.001			0.448
No	1115 (89.27)	134 (10.73)		279 (66.11)	143 (33.89)	
Yes	987 (95.83)	43 (4.17)		229 (63.26)	133 (36.74)	
**Peripheral nerve invasion**			<0.001			0.471
No	1389 (89.67)	160 (10.33)		351 (65.73)	183 (34.27)	
Yes	713 (97.67)	17 (2.33)		157 (62.80)	93 (37.20)	
**Lymphovascular invasion**			0.047			0.755
No	1588 (91.58)	146 (8.42)		402 (65.15)	215 (34.84)	
Yes	514 (94.31)	31 (5.69)		106 (63.47)	61 (36.53)	
**CEA**			0.022			0.033
Negative	1189 (91.18)	115 (8.82)		301 (67.95)	142 (32.05)	
Positive	875 (93.88)	57 (6.12)		197 (60.24)	130 (39.76)	
**AFP**			1			1
Negative	2041 (92.27)	171 (7.73)		64.66	35.34	
Positive	23 (95.83)	1 (4.17)		66.67	33.33	
**SCC**			1			0.337
Negative	1943 (92.30)	162 (7.70)		460 (64.16)	257 (35.84)	
Positive	121 (92.37)	10 (7.63)		38 (71.70)	15 (28.30)	
**NSE**			0.559			0.419
Negative	1352 (92.04)	117 (7.96)		295 (63.44)	170 (36.56)	
Positive	712 (92.83)	55 (7.17)		203 (66.56)	102 (33.44)	
**CA72-4**			<0.001			0.855
Negative	1733 (93.73)	116 (6.27)		396 (64.92)	214 (35.08)	
Positive	331 (85.53)	56 (14.47)		102 (63.75)	58 (36.25)	
**CA125**			0.479			0.179
Negative	1865 (92.46)	152 (7.54)		441 (63.82)	250 (36.18)	
Positive	199 (90.87)	20 (9.13)		57 (72.15)	22 (27.85)	
**CA15-3**			0.214			1
Negative	2062 (92.34)	171 (7.66)		497 (64.63)	272 (35.37)	
Positive	2 (66.67)	1 (33.33)		1 (100)	0 (0)	
**CA199**			1			0.006
Negative	1671 (92.32)	139 (7.68)		408 (67.22)	199 (32.78)	
Positive	393 (92.25)	33 (7.75)		90 (55.21)	73 (44.78)	
**FERR**			0.143			0.181
Negative	1942 (92.08)	167 (7.92)		467 (64.06)	262 (35.94)	
Positive	122 (96.06)	5 (3.94)		31 (75.61)	10 (24.39)	
**CYFRA21-1**			1			0.9
Negative	1525 (92.31)	127 (7.69)		366 (64.89)	198 (35.11)	
Positive	539 (92.29)	45 (7.71)		132 (64.08)	74 (35.92)	

Values presented are n (%) unless otherwise noted.

### KRAS Mutation Is Correlated With STMs and Clinicopathological Features

The STMs and clinicopathological features of the CRC patients are summarized in **Table 1** based on KRAS status. KRAS mutations were found to be more frequent in non-adenocarcinoma (43.56% vs. 32.30%, P = 0.005), right colon (42.04% vs. 32.09%, P = 0.009), well and moderately differentiated tumors (36.86% vs. 22.31%, P=0.003), positive CEA (39.76% vs. 32.05%, P = 0.033), and positive CA19-9 (44.78% vs. 32.78%, P = 0.006).

In addition to the established significance in metastatic colorectal cancer, it was also reported that KRAS mutations were correlated with a worse prognosis in stage II/III CRC (20, 21). Therefore, we further explored the correlation between KRAS status and STMs and clinicopathological features in stage II/III CRC (**Table 2**). The results demonstrated that KRAS mutation was highly associated with non-adenocarcinoma (45.21% vs. 32.54%, P = 0.003), right colon (43.04% vs. 32.47%, P = 0.008), well and moderately differentiated tumor (38.0% vs. 22.12%, P = 0.002), and positive CA 19-9 (45.10% vs. 33.46%, P = 0.011) but not associated with CEA (40.13% vs. 32.70%, P = 0.054).

**Table 2:** Associations of clinical characteristics with MMR and KRAS status among TNM (II/III) participants.

Characterization	MMR			KRAS		
	pMMR	dMMR	P-Value	Wild-Type	Mutant-Type	P-Value
**Age (years)**			0.023			0.172
<65	1255 (90.94)	125 (9.06)		317 (65.77)	165 (34.23)	
>=65	507 (94.24)	31 (5.76)		126 (60.00)	84 (40.00)	
**Gender**			0.976			0.162
Female	692 (91.78)	62 (8.22)		165 (60.66)	107 (39.34)	
Male	1070 (91.92)	94 (8.08)		278 (66.19)	142 (33.81)	
**Histology type**			0.021			0.003
non-adenocarcinoma	444 (89.33)	53 (10.66)		103 (54.79)	85 (45.21)	
adenocarcinoma	1318 (92.75)	103 (7.25)		340 (67.46)	164 (32.54)	
**Tumor size**			<0.001			0.212
<=4.6cm	1072 (96.75)	36 (3.25)		233 (61.80)	144 (38.20)	
>4.6cm	690 (85.19)	120 (14.81)		210 (66.67)	105 (33.33)	
**Tumor location**			<0.001			0.008
Right	411 (79.96)	103 (20.04)		131 (56.96)	99 (43.04)	
Left	1351 (96.23)	53 (3.77)		312 (67.53)	150 (32.47)	
**Degree of differentiation**			<0.001			0.002
Low	241 (87.32)	35 (12.68)		88 (77.88)	25 (22.12)	
Moderately and highly	1408 (93.87)	92 (6.13)		323 (62.00)	198 (38.00)	
**T stage**			0.077			0.732
I/II	82 (97.62)	2 (2.38)		16 (69.57)	7 (30.43)	
III/IV	1680 (91.60)	154 (8.40)		427 (63.83)	242 (36.17)	
**Harvested lymph nodes**			<0.001			0.976
<22	1229 (95.27)	61 (4.73)		267 (64.18)	149 (35.82)	
>=22	533 (84.87)	95 (15.13)		176 (63.77)	100 (36.23)	
**Lymph nodes metastasis**			<0.001			0.78
No	798 (87.40)	115 (12.60)		218 (64.69)	119 (35.31)	
Yes	964 (95.92)	41 (4.08)		225 (63.38)	130 (36.62)	
**Peripheral nerve invasion**			<0.001			0.56
No	1086 (88.51)	141 (11.49)		294 (64.90)	159 (35.10)	
Yes	676 (97.83)	15 (2.17)		149 (62.34)	90 (37.66)	
**Lymphovascular invasion**			0.028			1
No	1286 (91.01)	127 (8.99)		341 (64.10)	191 (35.90)	
Yes	476 (94.26)	29 (5.74)		102 (63.75)	58 (36.25)	
**CEA**			0.013			0.054
Negative	926 (90.52)	97 (9.48)		249 (67.30)	121 (32.70)	
Positive	807 (93.73)	54 (6.27)		185 (59.87)	124 (40.13)	
**AFP**			1			1
Negative	1713 (91.95)	150 (8.05)		431 (63.85)	244 (36.15)	
Positive	20 (95.24)	1 (4.76)		3 (75.00)	1 (25.00)	
**SCC**			1			0.379
Negative	1630 (91.99)	142 (8.01)		402 (63.41)	232 (36.59)	
Positive	103 (91.96)	9 (8.04)		32 (71.11)	13 (28.89)	
**NSE**			0.68			0.569
Negative	1124 (91.76)	101 (8.24)		260 (62.95)	153 (37.05)	
Positive	609 (92.41)	50 (7.59)		174 (65.41)	92 (34.59)	
**CA72-4**			<0.001			0.57
Negative	1439 (93.44)	101 (6.56)		346 (64.55)	190 (35.45)	
Positive	294 (85.47)	50 (14.53)		88 (61.54)	55 (38.46)	
**CA125**			0.424			0.133
Negative	1557 (92.18)	132 (7.82)		379 (62.85)	224 (37.15)	
Positive	176 (90.26)	19 (9.74)		55 (72.37)	21 (27.63)	
**CA15-3**			0.222			1
Negative	1731 (92.03)	150 (7.97)		433 (63.86)	245 (36.14)	
Positive	14 (66.67)	7 (33.33)		8 (61.5)	5 (38.5)	
**CA199**			1			0.011
Negative	1378 (91.99)	120 (8.01)		350 (66.54)	176 (33.46)	
Positive	355 (91.97)	31 (8.03)		84 (54.90)	69 (45.10)	
**FERR**			0.124			0.316
Negative	1628 (91.72)	147 (8.28)		407 (63.40)	235 (36.60)	
Positive	105 (96.33)	4 (3.67)		27 (72.97)	10 (27.03)	
**CYFRA21-1**			0.776			1
Negative	1253 (92.13)	107 (7.87)		309 (63.84)	175 (36.16)	
Positive	480 (91.60)	44 (8.40)		125 (64.10)	70 (35.90)	

Values presented are n (%) unless otherwise noted.

### DMMR Is Associated With STMs and Clinicopathological Features

The STMs and clinicopathological features of the CRC patients are summarized in **Table 1** according to MMR status. DMMR was more prone to occur in younger patients (8.72% vs. 5.26%, P = 0.008); in non-adenocarcinoma (10.59% vs. 6.81%, P = 0.005); and in tumors with larger diameters (14.58% vs. 3.60%, P < 0.001), right colon (20.28% vs. 3.67%, P < 0.001), low differentiation (13.20% vs. 5.86%, P < 0.001), deeper invasion (8.38% vs. 4.94%, P = 0.025), more harvested lymph nodes (14.90% vs. 4.62%, P < 0.001), fewer positive lymph nodes (10.73% vs. 4.17%, P < 0.001), no peripheral nerve invasion (10.33% vs. 2.33%, P < 0.001), no lymphovascular invasion (8.42% vs. 5.69%, P = 0.047), CEA-negative status (8.82% vs. 6.12%, P = 0.022) and CA72-4-positive status (14.47% vs. 6.27%, P < 0.001).

MMR status is an important factor when deciding whether to use adjuvant chemotherapy for patients with stage II CRC (5) and is a significant prognostic indicator in stage III CRC patients with recurrence after adjuvant chemotherapy (22). Therefore, we further analysed whether dMMR was associated with clinicopathological features and STMs in stage II/III CRC (**Table 2**). The results were similar to those for the whole CRC population, except for T stage, which was not associated with dMMR in stage II/III CRC.

### Predictive Value of STMs in Combination With Clinicopathological Features for KRAS Mutation

**Figure 3:**
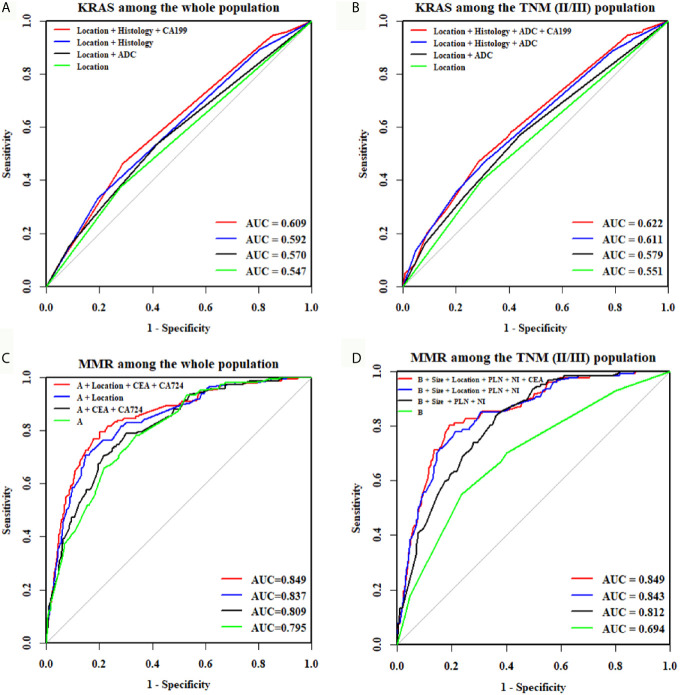
ROC curves of the combination of features for predicting KRAS or MMR status. The AUC of the KRAS mutation prediction rate was 0.609 in all participants **(A)** and 0.622 in TNM(II/III) participants **(B)**. The “A” in **(C, D)** means age + tumor size + degree of differentiation + harvested lymph nodes + positive lymph nodes + perineural invasion. The AUC, indicating the KRAS and MMR mutation prediction rate, was 0.849 in all participants **(C)** and 0.849 in TNM(II/III) participants **(D)**.

For the whole CRC population, univariate logistic regression analysis demonstrated that histology type, tumor location, degree of differentiation, and CEA and CA 19-9 levels were significantly associated with KRAS mutations (**Table 3**). When these predictive factors were subsequently assessed in the multivariate logistic regression, all except for non-adenocarcinoma and CEA remained highly significant. Therefore, right colon was found to be an independent predictor of KRAS mutations (OR, 1.550; P = 0.012), along with well and moderate differentiation (OR, 2.203; P = 0.001) and negative CA19-9 (OR, 1.600; P = 0.022). The predictive potential of these factors using ROC curves is shown in **Figure 3A**. When these three indexes were used together, the AUC was 0.609.

**Table 3:** Univariate and multivariate analyses of various predictive factors for KRAS status in all participants.

	Univariate Analysis OR (95% CI)	P-Value	Multivariate Analysis OR (95%CI)	P-Value
**Age (years)**		0.177		
<65	reference			
>=65	1.243 (0.905~1.705)			
**Gender**		0.43		
Female	reference			
Male	0.887 (0.658~1.196)			
**Histology type**		**0.004**		0.067
non-adenocarcinoma	reference		reference	
adenocarcinoma	0.618 (0.445~0.859)		0.696 (0.472~1.028)	
**Tumor size**		0.403		
<=4.6cm	reference			
>4.6cm	0.881 (0.653~1.185)			
**Tumor location**		**0.007**		**0.012**
Right	reference		reference	
Left	0.652 (0.477~0.890)		0.645 (0.458~0.909)	
**Degree of differentiation**		**0.002**		**0.001**
Moderately and highly	reference		reference	
Low	0.492 (0.306~0.768)		0.454 (0.278~0.721)	
**T stage**		0.190		
I/II	reference			
III/IV	1.354 (0.868~2.158)			
**Harvested lymph nodes**		0.782		
<22	reference			
>=22	1.044 (0.771~1.410)			
**Lymph nodes metastasis**		0.404		
No	reference			
Yes	1.133 (0.845~1.520)			
**Peripheral nerve invasion**		0.423		
No	reference			
Yes	1.136 (0.830~1.551)			
**Lymphovascular invasion**		0.687		
No	reference			
Yes	1.076 (0.751~1.532)			
**CEA**		**0.027**		0.321
Negative	reference		reference	
Positive	1.399 (1.038~1.885)		1.184 (0.848~1.650)	
**AFP**		0.918		
Negative	reference			
Positive	0.915 (0.126~4.718)			
**SCC**		0.270		
Negative	reference			
Positive	0.707 (0.370~1.283)			
**NSE**		0.376		
Negative	reference			
Positive	0.872 (0.642~1.180)			
**CA72-4**		0.783		
Negative	reference			
Positive	1.052 (0.729~1.508)			
**CA125**		0.144		
Negative	reference			
Positive	0.681 (0.399~1.125)			
**CA15-3**		0.980		
Negative	reference			
Positive	1.254 (0.652~1.674)			
**CA199**		**0.005**		**0.022**
Negative	reference		reference	
Positive	1.663 (1.168~2.364)		1.600 (1.068~2.394)	
**FERR**		0.137		
Negative	reference			
Positive	0.575 (0.264~1.152)			
**CYFRA21-1**		0.834		
Negative	reference			
Positive	1.036 (0.741~1.443)			

OR, odds ratio; 95% CI, 95% confidence interval.

Items were included in the multivariate analysis only when P value <0.05 in univariate analysis. The bold values represent significant difference (P < 0.05).

For stage II/III CRC, univariate logistic regression analysis showed that histology type, tumor location, degree of differentiation, and CEA and CA19-9 levels were significantly correlated with KRAS mutations (**Table 4**). In the multivariate logistic regression analysis, non-adenocarcinoma (OR, 1.553; P = 0.035), right colon (OR, 1.626; P = 0.008), well and moderate differentiation (OR, 2.227; P = 0.002) and positive CA19-9 (OR, 1.591; P = 0.030) were independent factors for predicting KRAS mutations. In addition, the AUC was 0.622 when these four indexes were used together (**Figure 3B**).

**Table 4:** Univariate and multivariate analyses of various predictive factors for KRAS status in TNM(II/III) participants.

	Univariate Analysis OR (95% CI)	P-Value	Multivariate Analysis OR (95%CI)	P-Value
**Age (years)**		0.147		
<65	reference			
>=65	1.281 (0.916~1.787)			
**Gender**		0.139		
Female	reference			
Male	0.788 (0.574~1.081)			
**Histology type**		0.020		0.035
non-adenocarcinoma	reference		reference	
adenocarcinoma	0.584 (0.415~0.824)		0.644 (0.427~0.972)	
**Tumor size**		0.185		
<=4.6cm	reference			
>4.6cm	0.809 (0.591~1.106)			
**Tumor location**		0.006		0.008
Right	reference		reference	
Left	0.636 (0.459~0.881)		0.615 (0.430~0.882)	
**Degree of differentiation**		0.002		0.002
Moderately and highly	reference		reference	
Low	0.463 (0.282~0.737)		0.449 (0.268~0.729)	
**T stage**		0.574		
I/II	reference			
III/IV	1.295 (0.545~3.410)			
**Harvested lymph nodes**		0.911		
<22	reference			
>=22	1.018 (0.741~1.397)			
**Lymph nodes metastasis**		0.720		
No	reference			
Yes	1.058 (0.776~1.445)			
**Peripheral nerve invasion**		0.505		
No	reference			
Yes	1.117 (0.806~1.545)			
**Lymphovascular invasion**		0.936		
No	reference			
Yes	1.015 (0.700~1.462)			
**CEA**		0.045		0.394
Negative	reference		reference	
Positive	1.379 (1.007~1.890)		1.165 (0.819~1.655)	
**AFP**		0.647		
Negative	reference			
Positive	0.589 (0.029~4.627)			
**SCC**		0.300		
Negative	reference			
Positive	0.704 (0.350~1.338)			
**NSE**		0.515		
Negative	reference			
Positive	0.899 (0.650~1.239)			
**CA72-4**		0.505		
Negative	reference			
Positive	1.138 (0.775~1.661)			
**CA125**		0.106		
Negative	reference			
Positive	0.646 (0.373~1.081)			
**CA15-3**		0.981		
Negative	reference			
Positive	1.232 (0.631~1.586)			
**CA199**		0.009		0.03
Negative	reference		reference	
Positive	1.634 (1.131~2.355)		1.591 (1.044~2.422)	
**FERR**		0.242		
Negative	reference			
Positive	0.641 (0.291~1.308)			
**CYFRA21-1**		0.949		
Negative	reference			
Positive	0.989 (0.697~1.395)			

OR, odds ratio; 95% CI, 95% confidence interval.

Items were included in the multivariate analysis only when P value <0.05 in univariate analysis.

### Predictive Value of STMs in Combination With Clinicopathological Features for MMR Status

For the whole CRC population, univariate logistic regression analysis showed that 12 potential predictors had a significant association with dMMR, including age, histology, tumor location, tumor size, degree of differentiation, T stage, harvested lymph nodes, positive lymph nodes, perineural invasion, lymphovascular invasion, CEA and CA 72-4 (**Table 5**). All these potential predictors were subsequently entered into a multivariate Cox regression analysis, and nine clinicopathological characteristics (age < 65 (OR, 1.923; P = 0.006), tumor size > 4.6 cm (OR, 2.646; P < 0.001), right colon (OR, 4.762; P < 0.001), poor differentiation (OR, 2.768; P < 0.001), harvested lymph nodes ≥ 22 (OR, 1.680; P = 0.012), no lymph node metastasis (OR, 2.924; P < 0.001), no perineural invasion (OR, 3.205; P < 0.001), negative CEA (OR, 1.667; P = 0.017) and positive CA 72-4 (OR, 1.901; P = 0.006) were finally selected as independent predictive factors for dMMR in the whole CRC patient cohort. When the nine criteria were used together, the AUC was 0.849 (**Figure 3C**).

**Table 5:** Univariate and multivariate analyses of various predictive factors for MMR status in all participants.

	Univariate Analysis OR (95% CI)	P-Value	Multivariate Analysis OR (95%CI)	P-Value
**Age (years)**		0.006		0.006
<65	reference		reference	
>=65	0.582 (0.388~0.848)		0.520 (0.322~0.815)	
**Gender**		0.914		
Female	reference			
Male	1.017 (0.745~1.397)			
**Histology type**		0.004		0.905
non-adenocarcinoma	reference		reference	
adenocarcinoma	0.617 (0.447~0.859)		1.029 (0.652~1.666)	
**Tumor size**		<0.001		<0.001
<=4.6cm	reference		reference	
>4.6cm	4.566 (3.279~6.448)		2.646 (1.721~4.123)	
**Tumor location**		<0.001		<0.001
Right	reference		reference	
Left	0.150 (0.108~0.206)		0.210 (0.138~0.316)	
**Degree of differentiation**		<0.001		<0.001
Moderately and highly	reference		reference	
Low	2.443 (1.645~3.566)		2.768 (1.724~4.398)	
**T stage**		0.020		0.477
I/II	reference		reference	
III/IV	1.760 (1.118~2.923)		0.808 (0.455~1.477)	
**Harvested lymph nodes**		<0.001		0.012
<22	reference		reference	
>=22	3.617 (2.647~4.965)		1.680 (1.120~2.525)	
**Lymph nodes metastasis**		<0.001		<0.001
No	reference		reference	
Yes	0.363 (0.252~0.512)		0.342 (0.209~0.545)	
**Peripheral nerve invasion**		<0.001		<0.001
No	reference		reference	
Yes	0.207 (0.120~0.334)		0.312 (0.167~0.552)	
**Lymphovascular invasion**		0.039		0.507
No	reference		reference	
Yes	0.656 (0.432~0.965)		1.212 (0.676~2.114)	
**CEA**		0.019		0.017
Negative	reference		reference	
Positive	0.674 (0.482~0.932)		0.600 (0.392~0.906)	
**AFP**		0.522		
Negative	reference			
Positive	0.519 (0.029~2.485)			
**SCC**		0.979		
Negative	reference			
Positive	0.991 (0.479~1.835)			
**NSE**		0.504		
Negative	reference			
Positive	0.893 (0.636~1.239)			
**CA72-4**		<0.001		0.006
Negative	reference		reference	
Positive	2.528 (1.789~3.535)		1.901 (1.195~2.979)	
**CA125**		0.401		
Negative	reference			
Positive	1.233 (0.736~1.965)			
**CA15-3**		0.143		
Negative	reference			
Positive	6.029 (0.279~63.251)			
**CA199**		0.963		
Negative	reference			
Positive	1.009(0.670~1.480)			
**FERR**		0.110		
Negative	reference			
Positive	0.477 (0.167~1.067)			
**CYFRA21-1**		0.989		
Negative	reference			
Positive	1.003 (0.697~1.417)			

OR, odds ratio; 95% CI, 95% confidence interval.

Items were included in the multivariate analysis only when P value <0.05 in univariate analysis.

For stage II/III CRC, all potential predictors were consistent with those for the whole CRC population, except for T stage, which was not associated with dMMR (**Table 6**). In the multivariate logistic regression, non-adenocarcinoma (P = 0.585), lymphovascular invasion (P = 0.354) and CA72-4 (P = 0.058) were found to be unrelated to dMMR, and the remaining eight indicators were identified as independent predictors for dMMR. When the eight criteria were used together, the AUC was 0.849 (**Figure 3D**).

**Table 6:** Univariate and multivariate analyses of various predictive factors for MMR status in TNM(II/III) participants.

	Univariate AnalysisOR (95% CI)	P-Value	Multivariate AnalysisOR (95%CI)	P-Value
**Age (years)**		0.019		0.025
<65	reference		reference	
>=65	0.614 (0.402~0.910)		0.572 (0.345~0.919)	
**Gender**		0.908		
Female	reference			
Male	0.981 (0.703~1.375)			
**Histology type**		0.017		0.585
non-adenocarcinoma	reference		reference	
adenocarcinoma	0.655 (0.464~0.933)		1.157 (0.696~1.991)	
**Tumor size**		<0.001		<0.001
<=4.6cm	reference		reference	
>4.6cm	5.179 (3.563~7.706)		2.892 (1.818~4.710)	
**Tumor location**		<0.001		<0.001
Right	reference		reference	
Left	0.157 (0.110~0.221)		0.231 (0.149~0.356)	
**Degree of differentiation**		<0.001		0.001
Moderately and highly	reference		reference	
Low	2.223 (1.455~3.328)		2.374 (1.435~3.879)	
**T stage**		0.066		
I/II	reference			
III/IV	3.758 (1.170~22.973)			
**Harvested lymph nodes**		<0.001		0.037
<22	reference		reference	
>=22	3.591 (2.570~5.052)		1.584 (1.028~2.449)	
**Lymph nodes metastasis**		<0.001		<0.001
No	reference		reference	
Yes	0.295 (0.202~0.423)		0.359 (0.218~0.578)	
**Peripheral nerve invasion**		<0.001		<0.001
No	reference		reference	
Yes	0.171 (0.096~0.284)		0.278 (0.143~0.503)	
**Lymphovascular invasion**		0.023		0.354
No	reference		reference	
Yes	0.617 (0.400~0.923)		1.320 (0.723~2.345)	
**CEA**		0.011		0.04
Negative	reference		reference	
Positive	0.639 (0.449~899)		0.637 (0.412~0.975)	
**AFP**		0.586		
Negative	reference			
Positive	0.571 (0.032~2.768)			
**SCC**		0.993		
Negative	reference			
Positive	1.003 (0.463~1.920)			
**NSE**		0.616		
Negative	reference			
Positive	0.914 (0.638~1.294)			
**CA72-4**		<0.001		0.058
Negative	reference		reference	
Positive	2.423 (1.678~3.461)		1.611 (0.974~2.614)	
**CA125**		0.349		
Negative	reference			
Positive	1.273 (0.746~2.063)			
**CA15-3**		0.153		
Negative	reference			
Positive	5.770 (0.267~60.573)			
**CA199**		0.989		
Negative	reference			
Positive	1.003 (0.564~1.495)			
**FERR**		0.095		
Negative	reference			
Positive	0.422 (0.128~1.024)			
**CYFRA21-1**		0.705		
Negative	reference			
Positive	1.073 (0.738~1.538)			

OR, odds ratio; 95% CI, 95% confidence interval.

Items were included in the multivariate analysis only when P value <0.05 in univariate analysis.

A summary of the AUCs of the individual and combined assessments used to predict mutation status is presented in **Table 7**.

**Table 7:** Summary of AUCs of the individual and combined assessments to predict gene mutation status.

	Whole Population		TNM(II/III) Subgroup	
	dMMR	KRAS (+)	dMMR	KRAS (+)
**Individual assessment**				
Age	0.548		0.582	
Histology type				0.554
Tumor size	0.68		0.696	
Tumor location	0.715	0.547	0.717	0.551
Degree of differentiation	0.57	0.545	0.561	0.551
Harvested lymph nodes	0.652		0.673	
Lymph nodes metastasis	0.613		0.666	
Peripheral nerve invasion	0.622		0.63	
CEA	0.546		0.554	
CA724	0.583			
CA199		0.544		0.544
**Combined assessment**	0.849	0.609	0.849	0.622

## Discussion

CRC is the third most common cancer in men and the second most common cancer in women worldwide, accounting for approximately 10% of all cancer-related deaths (23). To minimize the side effects of current treatments and achieve better prognosis, tremendous progress has been achieved in targeted therapy for CRC over recent decades. The status of KRAS and MMR was reported to be significantly correlated with the clinical outcomes of target therapy (7, 8, 12, 13, 24). For example, KRAS mutations make CRC less responsive to anti-EGFR monoclonal antibodies (7, 8), and dMMR makes CRC less likely to benefit from 5-FU-based chemotherapy (12, 13). However, the rate of KRAS and MMR detection was far below expected, mainly due to the following aspects: (1) a significant part of the population in developing counties cannot afford the high cost of gene detection; (2) the qualified clinical laboratory and professional team required for gene testing are not available in county-level hospitals as a result of significantly uneven distribution of medical resources in China; (3) Gene detection for all eligible patients would impose a substantial burden on the healthcare system. Therefore, establishing a convenient, non-invasive and cost-effective modality to identify appropriate candidates for genetic testing is urgently needed.

In this study, we explored the interrelationships among STMs, histopathological characteristics, and MMR and KRAS status using data from 2279 participants. Of the 784 patients tested for KRAS and 2279 patients tested for MMR status, KRAS mutations and dMMR were identified in 276 patients (35.20%) and 177 patients (7.77%), respectively. The discriminative ability of clinicopathological characteristics in combination with STMs was 0.609 for KRAS mutations and 0.849 for dMMR in the whole population. In addition, the combination of STMs and clinicopathological characteristics yielded an AUC of 0.622 for KRAS mutations and 0.849 for dMMR among TNM(II/III) participants.

Previous studies on the correlation between clinicopathological characteristics and KRAS mutations are controversial. Gao et al. (17) reported that no significant difference between KRAS mutations and tumor locations was observed, whereas Wilson et al. (25) and Julien et al. (26) supported that right-side CRC has a higher KRAS mutation rate. In our study, right colon (OR, 1.550; P = 0.012) and well and moderately differentiated tumors (OR, 2.203; P = 0.001) were independent predictive factors of KRAS mutations. STMs were reported to not be associated with KRAS mutations in several studies (20, 27, 28). However, negative CA199 was found to be significantly correlated with KRAS mutations in our study. Furthermore, the AUC was 0.609 when clinicopathological characteristics were combined with CA 19-9. The combination exceeds the discriminative ability of individual relevant factors, including right colon (AUC = 0.547), well and moderate differentiation (AUC = 0.545) and CA 19-9 (AUC = 0.544).

Previous studies have shown that MMR status was significantly correlated with clinicopathological characteristics of CRC (29–31), including tumor location, degree of differentiation, perineural invasion, and number of harvested lymph nodes, which are consistent with our results. In our study, younger age (OR, 1.923; P = 0.006), larger tumor (OR, 2.646; P < 0.001), and fewer positive lymph nodes (OR, 2.924; P < 0.001) were also independent predictive factors for dMMR. However, the existing evidence of the correlation between MMR status and STMs is controversial. Fan et al. (31) reported that dMMR was not associated with CEA, CA72-4, CA 242 and CA 19-9, but Schiemann et al. (32) reported that patients with high microsatellite instability had lower preoperative CEA serum levels than those with microsatellite stability. In our study, dMMR was significantly associated with negative CEA and positive CA72-4. When STMs were combined with clinicopathological characteristics, the AUC increased from 0.837 to 0.849. This result indicates that clinicopathological characteristics in combination with STMs can improve the discriminative ability of previously confirmed relevant factors, such as tumor location (AUC = 0.715), degree of differentiation (AUC = 0.57), perineural invasion (AUC = 0.622), and number of harvested lymph nodes (AUC = 0.652).

This study’s limitations deserve commentary. First, this was a nonrandomized retrospective analysis from a single centre, and as such, there were potential biases for comparison, such as patient inclusion and sample selection biases. Second, there is a lack of a validation group to further validate our results. Third, we did not evaluate the treatment response or perform a survival analysis according to clinical characteristics or serum tumor marker levels. However, our results demonstrated that clinicopathological characteristics in combination with STMs possessed a strong predictive power for KRAS and MMR status among CRC patients.

In conclusion, this is the largest retrospective study to investigate the interrelationship among KRAS mutations and dMMR, STMs and clinicopathological characteristics. KRAS mutations was significantly correlated with right colon, well and moderate differentiation and negative CA19-9. DMMR was significantly associated with younger age, larger tumors, right colon, poor differentiation, more harvested lymph nodes, fewer positive lymph nodes, no perineural invasion, negative CEA and positive CA72-4. The discriminative ability of clinicopathological characteristics combined with STMs reached 0.609 for KRAS mutations and 0.849 for dMMR. Radiomics signatures based on deep learning features have been used in previous studies to predict KRAS and MMR status and have resulted in remarkable accomplishments (31, 33–35). Therefore, for those CRC patients who could not undergo genetic testing, our findings will hopefully be integrated with radiomics or other markers to achieve a stronger discrimination ability of KRAS and MMR status.

## Data Availability Statement

The raw data supporting the conclusions of this article will be made available by the authors, without undue reservation.

## Ethics Statement

The studies involving human participants were reviewed and approved by Union Hospital, Tongji Medical College, Huazhong University of Science and Technology. Written informed consent to participate in this study was provided by the participants’ legal guardian/next of kin.

## Author Contributions

KC and TP conceived and designed the study. NZ, YC, JY, HL, KW, and JW collected and analysed data. NZ and YC wrote the paper. KC and TP reviewed and edited the manuscript. All authors contributed to the article and approved the submitted version.

## Funding

This study was supported by grants from the Science and Technology Department of Hubei Province (No.2018CFC884) and Free innovation pre-research fund and platform scientific research fund in 2019(02.03.2019-111).

## Conflict of Interest

The authors declare that the research was conducted in the absence of any commercial or financial relationships that could be construed as a potential conflict of interest.
